# A Review of Remediation Strategies for Diphenyl Ether Herbicide Contamination

**DOI:** 10.3390/toxics12060397

**Published:** 2024-05-29

**Authors:** Qingqing Fan, Yi Shen, Yong Yang, Qingming Zhang

**Affiliations:** Shandong Engineering Research Center for Environment-Friendly Agricultural Pest Management, College of Plant Health and Medicine, Qingdao Agricultural University, Qingdao 266109, China; f13954011774@163.com (Q.F.); 19853636589@163.com (Y.S.); yongyang@qau.edu.cn (Y.Y.)

**Keywords:** diphenyl ether herbicide, residual contamination, remediation technique, biochar

## Abstract

In agriculture, diphenyl ether herbicides are a broad-spectrum family of pesticides mainly used to control annual weeds in agriculture. Although diphenyl ether herbicides have a long-lasting effect in weed control, they can also be harmful to succeeding crops, as well as to the water and soil environment. Residual herbicides can also harm a large number of non-target organisms, leading to the death of pest predators and other beneficial organisms. Therefore, it is of great significance to control and remediate the contamination caused by diphenyl ether herbicide residues for the sake of environmental, nutritional, and biological safety. This review provides an overview of the techniques used for remediating diphenyl ether herbicide contamination, including biological, physical, and chemical remediation. Among these techniques, bioremediation, particularly microbial biodegradation technology, is extensively employed. The mechanisms and influencing factors of different remediation techniques in eliminating diphenyl ether herbicide contamination are discussed, together with a prospect for future development directions. This review serves as a scientific reference for the efficient remediation of residual contamination from diphenyl ether herbicides.

## 1. Introduction

**Figure 1:**
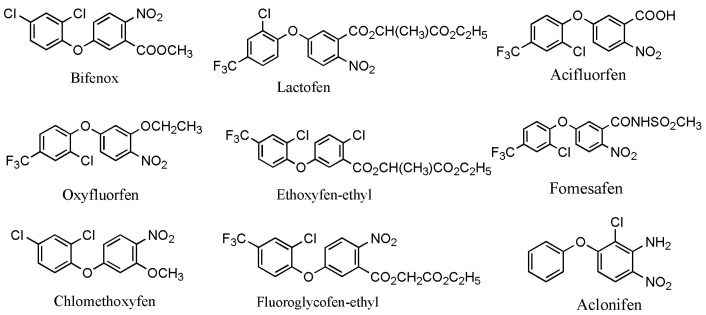
The main chemical structural formula of diphenyl ether herbicides [3].

Worldwide, pesticides play a significant role in controlling harmful pests and ensuring high and stable crop yields. However, the excessive use of pesticides has resulted in increasingly serious pollution issues; therefore, it is necessary to take specific measures to tackle these problems. The control of pesticide contamination is crucial for achieving sustainable agricultural development and environmental safety [1]. Among all types of pesticides, diphenyl ether herbicides are one of the most extensively utilized pesticides in the world [2]. Presently, there are a total of 11 varieties of diphenyl ether herbicides developed globally, mainly including fomesafen, lactofen, oxyfluorfen, acifluorfen, bifenox, ethoxyfen–ethyl, fluoroglycofen–ethyl, chlomethoxyfen, and aclonifen (Figure 1) [3]. Two other diphenyl ether herbicides, nitrofen and chlornitrofen, have been banned in numerous countries due to their high toxicity to mammals [4]. Diphenyl ether herbicides mainly enter the plant through the shoot apex and midrib, targeting protoporphyrinogen oxidase to inhibit chlorophyll synthesis and disrupt the cell membranes of sensitive plants. These herbicides are mainly applied to the soil surface as soil treatments. They primarily target the young shoots of annual weeds in soybean and peanut fields, with better effects on broadleaf weeds [5]. However, under adverse conditions (e.g., irregular temperatures and rainfall), the use of diphenyl ether herbicides to control broadleaf weeds in soybean fields can lead to phytotoxicity and inhibit soybean growth, in particular for some highly active and selective herbicides such as oxyfluorfen, fomesafen, and fluoroglycofen. Moreover, these herbicides can easily lead to phytotoxicity in the succeeding crops, even at low soil concentrations. The symptoms of phytotoxicity principally include leaf curling, burn-type lesions, and, in severe cases, the scorching of the entire leaf surface [6,7]. Diphenyl ether herbicides are relatively stable in the soil, with a long degradation half-life. For instance, the half-lives of fomesafen, oxyfluorfen, and acifluorfen in the soil were of 100–240 days, 30–40 days, and 14–60 days, respectively [8,9]. Subsequently, in recent years, some environmental problems resulting from the use of diphenyl ether herbicides have been frequently reported.

For example, oxyfluorfen has the potential to contaminate shallow groundwater in scenarios with low organic carbon content and coarse soil texture, which can significantly inhibit the activity of acetylcholinesterase in two fish species, namely the Nile tilapia and mosquito fish [10,11]. At higher concentrations (10^−1^–10^−3^ M), oxyfluorfen can potentially impact the development of human erythroid progenitor cells (BFU-E/CFU-E) and the synthesis of hemoglobin [12]. Lactofen can enter the soil through spraying, dust drift, or rainfall runoff, and exhibits high toxicity toward non-target organisms including mammals, fish, and aquatic invertebrates (e.g., large water fleas and diagonal water fleas) [13,14]. Bifenox, at concentrations of 0.5–0.75 µg/mL, can induce liver damage and toxic effects on the cardiovascular system in zebrafish embryos [15]. Additionally, some diphenyl ether herbicides, such as lactofen, contain asymmetric carbons and exhibit chirality, leading to enantioselectivity in their activity, environmental behavior, and toxicity. The research has demonstrated that the *R*-isomers of lactofen and acifluorfen exhibited higher toxicity than their *S*-isomers toward *Scenedesmus obliquus* and *Daphnia magna* [13,14]. Therefore, the toxicity difference between enantiomers of chiral diphenyl ether herbicides and specific contamination remediation should not be ignored.

In view of the above-mentioned adverse effects induced via the use of diphenyl ether herbicides, it is necessary to take relevant measures to reduce the residues of diphenyl ether herbicides in the environment and minimize their impact on the environment and non-target organisms. Previous studies have reported that the remediation methods for diphenyl ether herbicide contamination mainly include bioremediation, physical remediation, and chemical remediation. Based on this point, this review considers the development of diphenyl ether herbicide contamination remediation. The influencing factors and remediation mechanisms are also discussed. The objective of this review is to provide comprehensive information on the remediation of diphenyl ether herbicide contamination for scientific pollution control.

## 2. Bioremediation of Residual Contamination of Diphenyl Ether Herbicides

### 2.1. Progress in Bioremediation of Diphenyl Ether Herbicide Residue Contamination

Bioremediation technology is a sustainable and greener approach for the restoration of environmental pollution, especially soil pollution. It involves the use of plants, microorganisms, and other organisms to absorb and decompose harmful substances in the environment, thereby remediating the environment and removing pollutants. This technology can improve the pollution status of the agricultural soil environment to some extent [16]. It encompasses various techniques, mainly including phytoremediation and microbial degradation [17].

#### 2.1.1. Progress in Phytoremediation of Diphenyl Ether Herbicides Residue Contamination

Phytoremediation technology is an emerging soil remediation technique that offers several advantages, including easy operation, large-scale repair, minimal secondary pollution, low cost, and preservation of soil structure [17,18]. So far, there have been limited reports on using plants for remediating diphenyl ether herbicide contamination, with only the study of Wang et al. [19] investigating the enantioselective uptake and metabolism of the herbicide lactofen in the aquatic plant *Lemna minor*. It was found that *L. minor* exhibited a preference for the uptake and metabolism of S-lactofen, resulting in an accumulation approximately three times higher than that of R-lactofen in *L. minor*. Additionally, the metabolism rate of S-lactofen (T_1/2_ = 0.92 d) was significantly faster than that of R-lactofen (T_1/2_ = 1.55 d). However, phytoremediation technology has limitations in terms of the coverage depth and remediation period, and it cannot meet the demand for rapid remediation [20]. Therefore, further research and improvement are necessary.

#### 2.1.2. Progress in Microbial Degradation of Diphenyl Ether Herbicides Residue Contamination

Microorganisms can use organic pollutants as carbon source and energy and play an important role in the degradation of diphenyl ether herbicides [17]. Microorganisms possess diverse chemical abilities such as oxidation reduction, decarboxylation, deamination, and hydrolysis. Furthermore, microorganisms also have high reproductive ability and genetic variability, allowing their enzyme systems to rapidly adapt to environmental changes and showcasing metabolic diversity. Meanwhile, microorganisms are characterized by species diversity, wide distribution, fast reproduction, high surface area to volume ratio, and easy variability. In the environment, various natural substances, especially organic compounds, can almost always be degraded through the action of microorganisms [21]. Previous studies have reported that numerous microorganisms can degrade diphenyl ether herbicides. These microorganisms mainly include fungi, bacteria, and actinomycetes, and detailed information is provided in Table 1. Among all the diphenyl ether herbicides, fomesafen has been the most widely reported herbicide for contamination remediation using microorganisms. The reported microorganisms that have degradation ability mainly include *Aspergillus niger*, *Lysinibacillus*, *Shigella flexneri*, *Stenotrophomonas acidaminiphila*, *Pseudomonas zeshuii*, etc. [2,22,23,24,25,26,27,28,29,30,31,32,33,34,35,36,37,38,39]. In addition to the degradation of major diphenyl ether herbicides, some diphenyl ether derivatives have also been reported. For instance, Schmidt et al. [40] isolated a strain of *Sphingomonas* sp. SS3 that can degrade diphenyl ether and its mono-halogenated derivatives; Hiratsuka et al. [41] found that the white-rot fungus *Coriolus versicolor* can degrade diphenyl ether compounds through different metabolic pathways under lignin degradation conditions, and extracellular lignin-degrading enzymes do not participate in the initial steps of these metabolisms; Keum et al. [42] identified that *S. wittichii* RW1 is a highly effective diphenyl ether herbicide-metabolizing bacterium with broad substrate specificity.

### 2.2. Biodegradation Mechanisms of Diphenyl Ether Herbicides Residue Contamination

**Figure 2:**
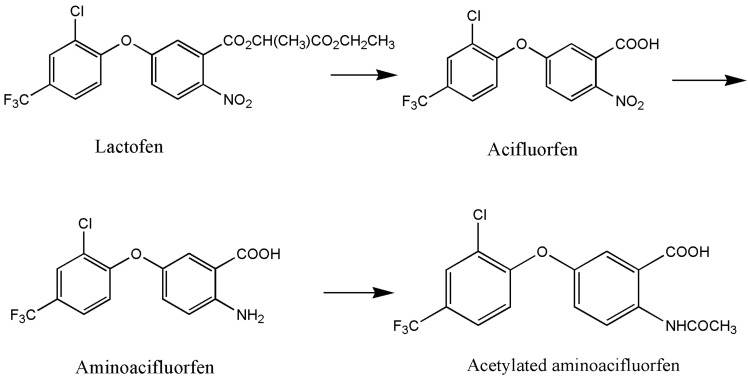
Degradation pathway of lactofen by *Bacillus* sp. YS-1 [34].

The research on biodegradation, particularly the microbial degradation technology of pesticides, has a long history. Effective microorganisms, including bacteria, fungi, and actinomycetes, have been successfully screened. Microorganisms exhibit diverse degradation mechanisms for pesticides, which can be categorized into enzymatic and non-enzymatic degradation [43]. In particular, enzymatic degradation is the primary pathway. In the presence of high concentrations of pesticide pollutants, microorganisms utilize enzymes to metabolize the specific toxic functional groups of pesticide molecules, thereby reducing or eliminating their toxicity. Hydrolases, esterase, and oxidoreductases are the typical enzymes involved in the degradation of chemical pesticides such as diphenyl ether herbicides [44]. For instance, Shang et al. [34] discovered that *Bacillus* sp. YS-1 degraded lactofen to three degradation products under the action of esterase *RapE* and *RhoE*. The degradation mechanism begins with the cleavage of two ester bonds in the side chain, resulting in the formation of acifluorfen. This is followed with the reduction in nitro groups to amino groups, leading to the formation of aminoacifluorfen. Finally, the amino group undergoes acetylation, resulting in the formation of acetylated aminoacifluorfen (Figure 2). Non-enzymatic degradation is another mechanism for the microbial degradation of chemical pesticides. It refers to the phenomenon whereby the molecular structure of the pesticide is degraded due to the change in pH value, the production of some auxiliary factors, and chemical substances produced in the process of microbial activity [45]. To date, there have been no reports on the non-enzymatic degradation mechanism of diphenyl ether herbicides.

The mechanism of phytoremediation can be categorized into three types: first, the direct adsorption of pesticides and their metabolites into the soil; second, the release of certain enzyme substances from plants into the rhizosphere, which directly degrades pesticides and their metabolites in the soil; third, the collaborative involvement of the plant rhizosphere and microorganisms in the process of pesticide decomposition. These three mechanisms frequently work together in plant remediation [46]. Organic pollutants in plants are primarily degraded through three steps: transformation, conjugation, and compartmentalization [47]. Transformation refers to the conversion of organic pollutants into metabolites with a lower hydrophobicity and toxicity through the action of plant enzymes. Conjugation involves the further combination of organic pollutants and metabolites generated in the first step with components in plants like glutathione, sugars, or amino acids, resulting in the formation of water-soluble conjugated metabolites through enzymatic reactions. Compartmentalization involves the transport of the low-toxicity-conjugated metabolites generated in the second step to the outside of the cell membrane via transporter proteins. These metabolites are then either stored in vacuoles or transferred to the extracellular matrix [48].

### 2.3. Factors Impacting the Bioremediation of Diphenyl Ether Herbicides Residual Contamination

#### 2.3.1. The Impact of Internal Factors

In relation to biodegradation, the intrinsic factors mainly include the concentration, inoculation amount, and culture time of the degrading strains. Each type of degrading bacteria has its own optimal conditions for exerting its degradation performance. For example, the optimal conditions for the degradation of *Lysinibacillus* sp. ZB-1, which was isolated from soil and capable of degrading fomesafen, were 28~36 °C, pH of 6.0, and the degradation rate attained 90% on the fifth day [24]. The optimal conditions for the degradation of *Shigella flexneri* FB5, which was also isolated from the soil, were 35~37 °C, pH of 6.0~7.0, and the degradation rate attained 88.32% within 96 h [27]. As for phytoremediation, the internal factors of phytoremediation mainly include the production of plant root exudates, the plant’s own metabolic activities, and the plant’s growth characteristics [47]. However, there is currently no research on the phytoremediation of diphenyl ether herbicide pollution.

#### 2.3.2. The Impact of Environmental Factors

Environmental factors include the physical and chemical properties of soil as well as the climatic conditions. Among these factors, the pH, temperature, and humidity of the soil exert a substantial impact on microbial degradation. The pH level directly impacts the types, community structure, and quantity of microbial communities. Additionally, different pH levels can alter the chemical form of diphenyl ether herbicides, indirectly impacting their degradation with microorganisms. Many microorganisms can only survive within specific pH ranges. Optimal pH levels facilitate microbial growth, whereas extreme pH conditions can be detrimental to their survival. The temperature affects degradation rates through control of the reaction rate of microbial enzymes. Both excessively high and low temperatures can affect reaction rates. For instance, in the study of Liang et al. [24] covering the microbial degradation of fomesafen, they discovered that the degradation rate of fomesafen varied significantly at different temperatures. Within 7 days, the degradation rates of fomesafen at 20 °C, 30 °C, and 37 °C were 38.8%, 81.2%, and 30.72%, respectively. This indicates that the microbial degradation rate of fomesafen is highest at 30 °C. Microbial growth requires adequate humidity and it has been shown that low humidity conditions can restrict the movement of microorganisms, while excessive humidity may block the capillaries between particles and limit the transport of oxygen. The optimal soil moisture content for microbial remediation is from 25% to 85% [49]. Therefore, it is crucial to investigate and establish the optimal conditions for the effective degradation of pollutants. Additionally, soil type, soil organic matter, different nutritional conditions, oxygen concentration, heavy metal ions, and other factors will also affect the degradation activity of microorganisms [7,50]. For example, previous studies have shown that the degradation of fomesafen is positively correlated with soil temperature, moisture content, and organic matter, but negatively correlated with soil pH [50].

In addition to the above-mentioned impact factors, the most significant limitation lies in the interactions between the introduced and indigenous microorganisms, including predators (viral lysis, protozoan grazers, and predatory bacteria), as well as competition for niches and nutrients. These interactions typically lead to the decrease in or suppression of foreign microorganisms following their introduction [51,52]. Additionally, a substantial reduction in or complete loss of the degrading capacity of the foreign degrading microorganisms may be observed when they are introduced into pesticide-polluted environments, which also represents a significant limiting factor [33,53]. Therefore, there are still important challenges in using degrading microorganisms for the in situ remediation of pesticide-contaminated environments.

## 3. Physical Remediation of Diphenyl Ether Herbicide Residual Contamination

### 3.1. Progress in Physical Remediation of the Diphenyl Ether Herbicide Residual Contamination

Physical remediation methods refer to the application of physical techniques to remove pollutants from the environment. There are three principal types of treatment methods: adsorption, steam extraction, and thermal desorption. In steam extraction technology, pipelines are used to heat the soil, causing moisture to evaporate and thereby carry away residual pesticides. On the other hand, thermal desorption technology directly heats the soil to volatilize the residual pesticides [20]. Physical remediation through adsorption, particularly using biochar as an adsorbent, is a more effective and widely employed method compared to steam extraction and thermal desorption.

Biochar is a stable and carbon-rich porous material prepared via the pyrolysis of biomass. Biochar is a porous, carbonaceous solid produced using the thermochemical conversion of organic materials in a low-oxygen atmosphere. Biochar’s physicochemical properties make it suitable for safe and long-term storage in the environment, as well as for improving soil properties. Additionally, biochar itself, as a recalcitrant form of carbon, serves as a long-term carbon storage medium [54]. As a new material, it possesses high aromatic qualities, excellent adsorption, high stability, is cost-effective, and environmentally friendly. It can be used to immobilize pollutants, and minimize soil pollution risks to a significant extent. Consequently, it has proved to be an outstanding material for soil remediation purposes [55,56]. The research has shown that adding biochar materials to the soil can effectively adsorb diphenyl ether herbicides, enhance the activity of microorganisms in the soil, and reduce the risk of soil contamination. For example, Wu et al. [57] used five types of biochar (peanut biochar, chestnut biochar, bamboo biochar, maize straw biochar, and rice hull biochar) to adsorb oxyfluorfen and found that the rice hull biochar has the best optimal absorption effect for oxyfluorfen. When the rice hull-derived biochar was added into three different types of soil, the results showed that its application enhanced the adsorption of oxyfluorfen, and with the increase in biochar content, the adsorption capacity of the biochar–soil system also increased. Furthermore, biochar can directly or indirectly enhance the activity of soil microorganisms, improve soil nutrient retention capacity and biological effectiveness, improve the soil quality and its physicochemical properties, and indirectly influence the soil structure to improve plant growth and nutrient cycling. During the research on biochar and modified biochar adsorption of oxyfluorfen, Wu also identified that biochar not only plays a role in adsorption, but can also increase the diversity and abundance of soil microbial communities. In order to overcome the shortcomings of the long processing time, shallow soil layer, and fluctuating effects of biochar methods, the methods deploying biochar have been strengthened, including gas-modified biochar, acid-modified biochar, alkali-modified biochar, and metal-modified biochar [48]. The current research results indicate that the application of biochar, as a powerful and high-performance soil pollution remediation material, not only reduces the health risks associated with pesticides, but also reduces the environmental risks and increases the ecological and agricultural benefits [58,59].

### 3.2. Physical Remediation Mechanism of Diphenyl Ether Herbicide Residual Contamination

**Figure 3:**
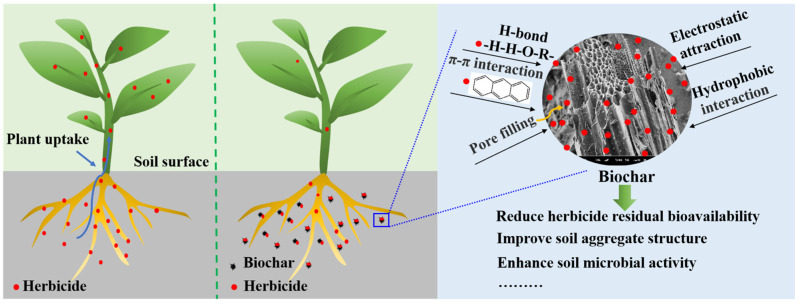
The remediation mechanism of herbicide-contaminated environment using biochar [60,61].

In this review, we have only discussed the mechanism of adsorption. As is widely recognized, adsorption plays a crucial role in determining the fate of pesticides in the soil. Adsorption is a physical and chemical process that involves the retention of pesticide molecules on solid surfaces through various types of bonding, including hydrogen bonding, ionic bonding, covalent bonding, and Van der Waals forces. These interactions occur between charged sites on soil colloids [60]. The adsorption process of biochar on pesticides, including diphenyl ether herbicides, can be categorized into both physical and chemical adsorption (Figure 3). Pore volume, pore size, and specific surface area are factors affecting physical adsorption [61]. Chemical adsorption involves intermolecular π-π conjugation, hydrogen bonding, hydrophobic interaction, and electrostatic attraction between the pesticides and the organic molecules from biochar [60]. In this interaction, pesticide molecules can bond with a variety of functional groups [62]. The surface hydrophilic groups, overall properties, and porous surface of biochar have an impact on the adsorption of hydrophilic compounds and hydrophobic compounds [63]. The current research indicates that biochar obtained directly from biomass pyrolysis has poor surface functionality, low porosity, a low surface area, and very limited functional groups such as hydroxyl and carbonyl [64]. Although these drawbacks limit the widespread application of biochar, its surface functionality and porosity can be adjusted through technological advancements. Therefore, the potential for biochar application remains extensive. The surface properties and pore structure of biochar can be adjusted through chemical reactions, such as metal salt solution impregnation, oxidation, amination, sulfonation, and surface doping. These reactions allow for the loading of metal ions and grafting of functional groups, which in turn facilitate the functionalization of biochar’s surface properties [54].

### 3.3. Impact Factors of Physical Remediation (Adsorption)

The adsorption of pesticides onto remediation materials, such as biochar, is influenced by various factors. These factors include the characteristics of biochar (aromaticity, carbon content, porosity, surface area, surface functional groups, electrostatic attraction), the properties of the pesticide (hydrophilicity/hydrophobicity), and the properties of the soil (organic matter content, pH) [54,59]. The source of biochar raw materials and the temperature of pyrolysis are crucial factors that influence the adsorption capacity [65,66]. Additionally, the soluble organic matter present in environmental matrices can compete with chemical pollutants through occupation of the adsorption sites on the biochar surface and blocking its porous structure, thereby impacting the adsorption capacity [67]. Furthermore, natural aging processes can also influence the adsorption effectiveness of biochar [68,69]. As for diphenyl ether herbicides, Wu et al. [57] illustrated that the adsorption coefficient (Kf) values of oxyfluorfen in five biochars (peanut, chestnut, bamboo, maize straw, and rice hull) at the same preparation temperature (300 °C) were significantly different. Among them, the adsorption capacity of rice hulls was the strongest, and the adsorption capacity of rice hull biochar increased with the increase in the pyrolysis temperature. In addition, they found that the specific surface area (SSA) of biochar was positively correlated with Kf [57]. Khorram et al. [70] compared the adsorption properties of six biochars (rice straw, softwood, hardwood, nut shell, coconut shell, and bamboo) on another diphenyl ether herbicide, fomesafen. They also found that the adsorption capacity of biochar on fomesafen is most likely controlled with the SSA and the dissolved organic carbon contents of biochar. Therefore, it is important to design a suitable material according to the characteristics of the pesticides for an increased adsorption effect.

## 4. Chemical Remediation of Diphenyl Ether Herbicide Residual Contamination

### 4.1. Progress in Chemical Remediation of the Diphenyl Ether Herbicide Residual Contamination

The use of chemical technology to remediate the residues of herbicides is also an important method. The chemical remediation methods mainly include chemical oxidation and leaching methods. In the field of chemical oxidation methods, the research focus is on advanced oxidation methods, including ozone catalytic oxidation, catalytic wet oxidation, photocatalytic oxidation, and electrocatalytic oxidation, among others. As for the leaching method, it can be divided into in situ remediation and ex situ remediation based on the location of soil treatment [20].

As for the chemical remediation of diphenyl ether herbicide contamination, current research is mostly focused on photocatalysis. For example, Xin [71] prepared a series of Cu or Sn-TiO_2_ photocatalysts using the sol–gel method. It was found that compared to traditional TiO_2_, the modified catalyst is not only inexpensive and easy to obtain but also broadens the light response range of TiO_2_, improves its quantum efficiency, and effectively enhances the degradation efficiency of acifluorfen. Zhang et al. [72] investigated the degradation of wastewater containing the herbicide acifluorfen through the preparation of doped and modified TiO_2_ photocatalysts. In addition, they explored the factors influencing the efficiency of photocatalytic degradation. Liang et al. [73] aimed to accelerate the degradation of diphenyl ether herbicides in a visible light environment. They utilized corn cobs as the source of carbon quantum dots and modified them using nitrogen carbon to synthesize a non-metallic photocatalyst. Then, the degradation efficiency of fomesafen, oxyfluorfen, and acifluorfen under the action of this catalyst was studied. In order to provide basic data support for the remediation and control of such agricultural chemical pollution, the toxicity of the photodegradation products was also evaluated using a corn seed germination model. The results of this study confirm that the stability and toxicity of diphenyl ether herbicides in a visible light environment vary significantly with different substituents. Moreover, this study verified that fomesafen is the most easily photodegraded pesticide but has the most significant toxicity; meanwhile, acifluorfen has the lowest photodegradation rate but is the safest in terms of toxic effect. Gaggara et al. [74] prepared ZnO nanoparticles using the sol–gel method and investigated the photocatalytic performance of ZnO nanoparticles on fomesafen, at a pH level of 4, 7, and 9. The effects of ultraviolet light, aeration, and the addition of oxidants to the reaction mixture were also studied. The results show that the degradation rate was accelerated in the presence of sunlight and nanomaterials. Acosta-Santoyo et al. [75] evaluated the electrochemical dewatering of sludge generated after the coagulation of wastewater contaminated with oxyfluorfen. This process not only reduces the volume of sludge but also helps to degrade the oxyfluorfen contained in the resulting cake with electrolysis and using boron-doped diamond anodes. In three consecutive stages, the application of an electric field during the electrochemical phase enabled the electrolytic degradation of the oxyfluorfen. Dos Santos et al. [76] studied the surfactant-assisted soil washing (SASW) method for treating soil contaminated with oxyfluorfen, and then used photoelectrochemical oxidation to treat the washing solution produced. The results showed that surfactant-assisted soil washing can effectively remove oxyfluorfen from the soil and transfer it to the washing solution. It was also found that diamond electrode photocatalysis is a very effective treatment technology. The combination of ultraviolet irradiation and electrolysis significantly improves the removal efficiency of oxyfluorfen, while having little effect on the removal efficiency of surfactants.

### 4.2. Chemical Remediation Mechanism of Diphenyl Ether Herbicide Residual Contamination

**Figure 4:**
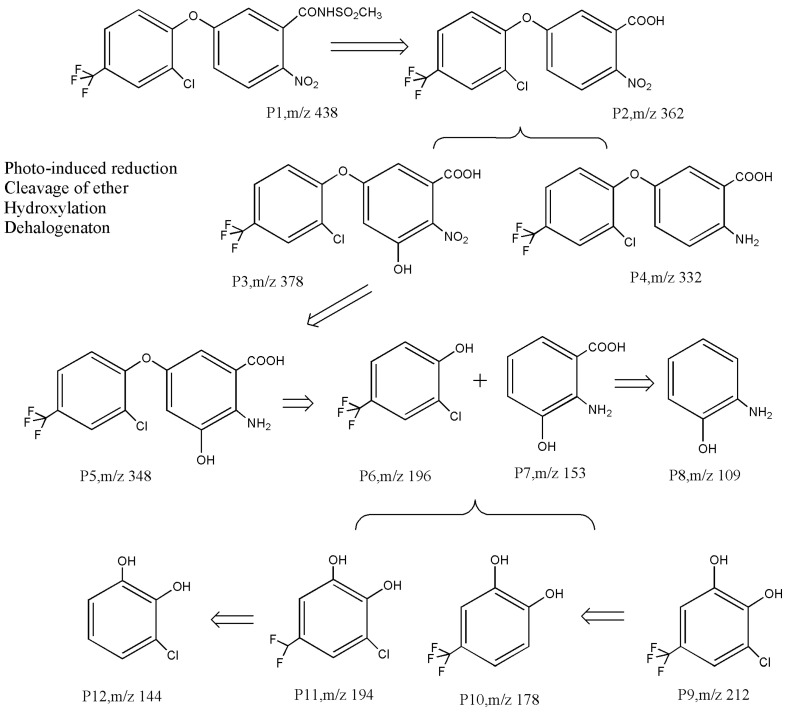
Photocatalytic degradation pathway of acifluorfen [73].

There is very little research on the photodegradation remediation process of diphenyl ether compounds. Chakraborty et al. [77] reported that the photodegradation products of oxyfluorfen may undergo ether bond cleavage and dehalogenation pathways. In the process of using TiO_2_ to degrade acifluorfen, Xin [71] also confirmed the cleavage of ether bonds, dehalogenation, hydroxylation, and other products. As shown in Figure 4, Liang et al. [73] inferred that the photocatalytic degradation process of diphenyl ether herbicides includes at least three pathways: (1) hydrolysis, dehalogenation, and nitro reduction; (2) ether bond cleavage; and (3) hydroxylation. Taking the most easily degradable fomesafen P1 as an example, it undergoes nucleophilic and free radical attacks to form the intermediate products P2–P5 through amide hydrolysis and nitro reduction, followed with ether bond cleavage to yield the phenoxyl intermediates P6 and P7. Electrophilic- and free radical-driven hydroxylation and dehalogenation reactions may occur on the C-H, C-Cl, and C-F bonds on the aromatic ring [78], resulting in intermediate products such as P8–P12. These primary aromatic hydrocarbons are more easily degraded until they mineralize into CO_2_ and H_2_O.

### 4.3. Impact Factors of Chemical Remediation

Photocatalytic degradation affecting factors for diphenyl ether herbicides mainly include light intensity, analyst characteristics and dosage, and reaction conditions. For example, Xin, Zhang, and others [71,72] made improvements to TiO_2_; however, through experiments, it was discovered that the improved TiO_2_ was not only affected by the dosage of the catalyst, but also by various factors such as light exposure time, solution pH value, some commonly used anionic and cationic species (Cl^−^, NO^3−^, SO_4_^2−^, PO_4_^3−^, K^+^, Ca^2+^, and Cu^2+^) in agricultural production, and the initial concentration of the degradation target in photocatalytic degradation. Gaggara et al. [74] used ZnO/Na_2_S_2_O_8_ as a catalyst/oxidant to degrade fomesafen photo-catalytically under ultraviolet light irradiation. The optimal dosage of the catalyst was found to be 100 mg/L. The half-lives of fomesafen with ZnO/Na_2_S_2_O_8_ in the three different pH solutions were 16.03 h, 15.56 h, and 11.93 h, respectively. The reaction rate was faster under alkaline conditions, and slower under acidic and neutral conditions. Acosta-Santoyo et al. [75] found that the design of an electrochemical dewatering cell has a significant impact on the process performance during the dewatering of oxyfluorfen-contaminated sludge. When the cathode is placed upstream and the anode is placed downstream, the electrolytic removal efficiency of oxyfluorfen is higher. Therefore, it is crucial to design inexpensive catalyst materials with excellent performance and develop mild reaction conditions for the degradation of diphenyl ether herbicides.

## 5. Conclusions and Perspectives

In this review, different remediation technologies for diphenyl ether herbicide contamination were discussed. Among all remediation technologies, the use of degrading strains to clean up soil contaminated with diphenyl ether herbicides has received widespread attention. However, microbial remediation also faces some challenges. For example, the survival, competition with native microorganisms, and degradative activity of inoculated strains are critical factors that limit the efficiency of the bioremediation process. Despite the high adaptability of microorganisms to diverse environments, the low survival and activity of individual bioaugmentation strains often result in decreased efficiency or failure of remediation. In summary, each method, whether biological, physical, or chemical, has its limitations when used alone for the remediation of diphenyl ether herbicides. Therefore, the future research trend for the remediation of diphenyl ether herbicides lies in the combined application of multiple methods to compensate for or enhance the remediation effect. Additionally, since many technologies are still at the laboratory or small-scale field trial stage, further research is needed to ensure their effectiveness and stability for large-scale use and promotion. In practical applications, it is especially important that the selection of a suitable remediation scheme should be based on a comprehensive evaluation of different remediation objects, residual pesticide types, and economic factors.

## Figures and Tables

**Table 1:** Microbial species, sources, and degradation characteristics of degradable diphenyl ether herbicides.

Degrading Strains	Source	Herbicide	Concentration (mg/L)	Degradation Rate (%)	Degradation Mechanism	References
**Bacteria**						
*Pseudomonas mendocina* FB8	soil	Fomesafen	500	86.75 (37 °C, pH 7.0, 96 h)	Mineralization	[22]
*Pseudomonas* sp. TB-2	soil	Fomesafen	10	96.00 (35 °C, pH 7.0, 72 h)	Co-metabolism	[23]
*Lysinibacillus* sp. ZB-1	soil	Fomesafen	50	81.32 (30 °C, 7 d)	Mineralization	[24]
*Bacillus* sp. FE-1	soil	Fomesafen	10	82.90 (35 °C, pH 7.0, 14 h)	Mineralization	[25]
*Klebsiella* sp. F-12	soil	Fomesafen	100	80.00 (35 °C, pH 6.0, 2 d)	Mineralization	[26]
*Klebsiella* sp. FB9	soil	Fomesafen	500	48.32 (37 °C, pH 7.0, 96 h)	Mineralization	[27]
*Shigella* sp. FB5	soil	Fomesafen	500	88.32 (37 °C, pH 7.0, 96 h)	Mineralization	[27]
*Stenotrophomonas acidaminiphila* BX3	Sewage	Fomesafen	100	80.00 (30 °C, pH 6.0~7.0, 5 d)	Mineralization	[28]
*Sinorhizobium* sp. W16	soil	Fomesafen	5	69.00 (30 °C, 7 d)	Mineralization	[29]
*Pseudomonas zeshuii* BY-1	soil	Fomesafen	50	88.70 (3 d)	Mineralization	[30]
*Pseudomonas* sp. YF1	soil	Fluoroglycofen	200	80.00 (30 °C, pH 7.0, 7 d)	Mineralization	[31]
*Lysinibacillus* sp. ZB-1	soil	Fluoroglycofen	50	86.40 (30 °C, pH 7.0, 7 d)	Mineralization	[24]
*Pseudomonas citronellolis* DK-3	Sludge	Acifluorfen	100	97.20 (30 °C, pH 7.0–8.0, 120 h)	Mineralization	[32]
*Lysinibacillus* sp. ZB-1	soil	Lactofen	50	60.40 (30 °C, 7 d)	Mineralization	[24]
*Bacillus* sp. Za	soil	Lactofen	50	94.80 (40 °C, pH 7.0, 4 d)	Co-metabolism	[33]
*Bacillus* sp. YS-1	soil	Lactofen	50	97.60 (pH 8.0, 15 h)	Mineralization	[34]
*Azotobacter chroococcum*	soil	Oxyfluorfen	240	60.00 (29 °C, pH 7.0, 7 d)	Mineralization	[35]
*Chryseobacterium aquifrigidense* R-21	soil	Oxyfluorfen	50	92.10 (33.4 °C, pH 6.9, 5 d)	Mineralization	[2]
**Fungi**						
*Aspergillus niger* S7	soil	Fomesafen	100	90.00 (28–36 °C, pH 6.0, 5 d)	Mineralization	[36]
*Aspergillus flavus* TZ1985	soil	Fomesafen	40	92.13 (25 °C, pH 7.0, 5 d)	Mineralization	[37]
*Aspergillus jensenii* FF1	soil	Fomesafen	600	21.03 (30 °C, pH 7.0, 7 d)	Mineralization	[38]
*Penicillium dipodomyicola* FF2	soil	Fomesafen	600	15.74 (30 °C, pH 7.0, 7 d)	Mineralization	[38]
*Rhizopus oryzae* FF3	soil	Fomesafen	600	11.88 (30 °C, pH 7.0, 7 d)	Mineralization	[38]
**Actinomycetes**						
*Mycobacterium* sp. MBWY-1	Sludge	Fluoroglycofen	100	91.60 (30 °C, pH 6.0–7.0, 96 h)	Mineralization	[39]

## Data Availability

The data supporting this study’s findings are available from the corresponding author upon reasonable request.
